# Pharmacological inhibition of Hippo pathway, with the novel kinase inhibitor XMU‐MP‐1, protects the heart against adverse effects during pressure overload

**DOI:** 10.1111/bph.14795

**Published:** 2019-10-08

**Authors:** Efta Triastuti, Ardiansah Bayu Nugroho, Min Zi, Sukhpal Prehar, Yulia Suciati Kohar, Thuy Anh Bui, Nicholas Stafford, Elizabeth J. Cartwright, Sabu Abraham, Delvac Oceandy

**Affiliations:** ^1^ Division of Cardiovascular Sciences, Faculty of Biology, Medicine and Health The University of Manchester, Manchester Academic Health Science Centre Manchester UK; ^2^ Department of Pharmacy, Faculty of Medicine Universitas Brawijaya Malang Indonesia; ^3^ Department of Biochemistry, Faculty of Medicine YARSI University Jakarta Indonesia

## Abstract

**Background and Purpose:**

The Hippo pathway has emerged as a potential therapeutic target to control pathological cardiac remodelling. The core components of the Hippo pathway, mammalian Ste‐20 like kinase 1 (Mst1) and mammalian Ste‐20 like kinase 2 (Mst2), modulate cardiac hypertrophy, apoptosis, and fibrosis. Here, we study the effects of pharmacological inhibition of Mst1/2 using a novel inhibitor XMU‐MP‐1 in controlling the adverse effects of pressure overload‐induced hypertrophy.

**Experimental Approach:**

We used cultured neonatal rat cardiomyocytes (NRCM) and C57Bl/6 mice with transverse aortic constriction (TAC) as in vitro and in vivo models, respectively, to test the effects of XMU‐MP‐1 treatment. We used luciferase reporter assays, western blots and immunofluorescence assays in vitro, with echocardiography, qRT‐PCR and immunohistochemical methods in vivo.

**Key Results:**

XMU‐MP‐1 treatment significantly increased activity of the Hippo pathway effector yes‐associated protein and inhibited phenylephrine‐induced hypertrophy in NRCM. XMU‐MP‐1 improved cardiomyocyte survival and reduced apoptosis following oxidative stress. In vivo, mice 3 weeks after TAC, were treated with XMU‐MP‐1 (1 mg·kg^−1^) every alternate day for 10 further days. XMU‐MP‐1‐treated mice showed better cardiac contractility than vehicle‐treated mice. Cardiomyocyte cross‐sectional size and expression of the hypertrophic marker, brain natriuretic peptide, were reduced in XMU‐MP‐1‐treated mice. Improved heart function in XMU‐MP‐1‐treated mice with TAC, was accompanied by fewer TUNEL positive cardiomyocytes and lower levels of fibrosis, suggesting inhibition of cardiomyocyte apoptosis and decreased fibrosis.

**Conclusions and Implications:**

The Hippo pathway inhibitor, XMU‐MP‐1, reduced cellular hypertrophy and improved survival in cultured cardiomyocytes and, in vivo, preserved cardiac function following pressure overload.

**Abbreviations**
ANP: atrial natriuretic peptide
BNP: brain natriuretic peptide, (B‐type natriuretic peptide)
HW/TL: heart weight/tibia length
Lats: large tumour suppressor kinase
MOB1: MOB kinase activator 1
Mst: mammalian Ste‐20 like kinase
NRCM: neonatal rat cardiomyocytes
Sav1: Salvador homologue 1
TAC: transverse aortic constriction
YAP: yes‐associated protein

## 1 INTRODUCTION

Heart failure has become a global health problem with projections of increasing prevalence and rising economic burden in the next decade (Cook, Cole, Asaria, Jabbour, & Francis, 2014; Mozaffarian et al., 2015). A number of processes are believed to contribute significantly to the pathological mechanisms of heart failure, such as loss of cardiac cells through apoptosis or necrosis, enlargement of cardiac cells (hypertrophy), and fibrosis. Together, these processes may lead to adverse cardiac remodelling, which if left untreated can eventually progress to heart failure (Burchfield, Xie, & Hill, 2013).

One of the major causes of adverse cardiac remodelling is chronic pressure overload. At the initial stage of remodelling, hypertrophic growth is regarded as an adaptive response to reduce stress on the ventricular wall (Hill & Olson, 2008). However, in the long term, hypertrophy is likely to produce detrimental effects since a number of clinical data have shown strong associations between cardiac hypertrophy and the incidence of heart failure (Yang, Negishi, Otahal, & Marwick, 2015).

Cardiac pressure overload through the increase of mechanical wall stress and activation of neurohormonal factors induces a number of molecular signalling pathways that are involved in promoting cardiomyocyte growth (Heineke & Molkentin, 2006). On the other hand, pressure overload also induces significant cardiomyocyte death (Hein et al., 2003). Apoptosis appears to play a major role in mediating cell death since prolonged pressure overload triggers expression of pro‐apoptotic proteins and reduces the level of anti‐apoptotic molecules (Kuster et al., 2005). This has prompted an idea that modulation of both hypertrophic and apoptotic signals in the heart during pressure overload will produce beneficial effects.

The Hippo signalling pathway is a kinase cascade known to control organ size through regulation of proliferation and apoptosis. Importantly, it has been associated with a number of key pathophysiological processes in the heart, such as regulation of cardiomyocyte apoptosis (Matsui et al., 2008; Yamamoto et al., 2003), cardiac hypertrophy (Zi et al., 2014), autophagy (Maejima et al., 2013), and cardiomyocyte proliferation (Heallen et al., 2013). The core components of this pathway include http://www.guidetopharmacology.org/GRAC/ObjectDisplayForward?objectId=2225 and http://www.guidetopharmacology.org/GRAC/ObjectDisplayForward?objectId=2219 (Mst1 and Mst2), http://www.guidetopharmacology.org/GRAC/ObjectDisplayForward?objectId=1515 and http://www.guidetopharmacology.org/GRAC/ObjectDisplayForward?objectId=1516 (Lats1 and Lats2), and the adaptor molecules Salvador homologue 1 (Sav1) and MOB kinase activator 1 (MOB1). Upon activation, Mst1/2 phosphorylate Lats1/2. The latter will phosphorylate yes‐associated protein (YAP), the main effector of the pathway, which results in its cytoplasmic retention and inactivation. In the heart, the pathway is thought to be activated by a variety of upstream stress signals including ROS, mechanical stress, and GPCR signalling (Zhou, Li, Zhao, & Guan, 2015). Notably, the expression and activation of core components of the Hippo pathway such as YAP, Lats, and Salv were significantly elevated in patients with heart failure (Leach et al., 2017) and we have previously shown expression of Mst2 was increased in a mouse model of pressure overload hypertrophy (Zi et al., 2014). These results indicate that in the heart, Hippo pathway components can be triggered following pathological stimuli, and thus, targeting the Hippo pathway may have clinical relevance.

The two main core kinases of the Hippo pathway, the Mst1 and Mst2, have been implicated in the regulation of cell apoptosis (Galan & Avruch, 2016). Overactivation of Mst1 in cardiomyocytes led to severe cardiomyopathy due to induction of apoptosis (Yamamoto et al., 2003). Interestingly, the second isoform of this enzyme (i.e., Mst2) has been implicated in cardiac hypertrophy (Zi et al., 2014). This indicates that inhibition of Mst1 and Mst2 in the heart might be beneficial through modulation of hypertrophy and reduction of apoptosis. As pharmacological therapy that directly targets intra‐cardiac molecules to control adverse remodelling is currently limited (Xie, Burchfield, & Hill, 2013), targeting Mst1/2 could become a promising approach to address this problem.

A recent study (Fan et al., 2016) described a novel benzenesulfonamide, XMU‐MP‐1, that selectively inhibited Mst1 and Mst2 with IC_50_ values of 71 and 38 nM, respectively. In vivo, XMU‐MP‐1 treatment induced regeneration of the liver and intestine after injury in mice, through the modulation of the Hippo pathway (Fan et al., 2016). However, the effect of XMU‐MP‐1 treatment in the heart during pathological conditions has not been investigated. Thus, in this study, we used both in vitro and in vivo models to investigate the effects of the Mst1/2 inhibitor XMU‐MP‐1 on pathological cardiac hypertrophy.

## 2 METHODS

### 2.1 Isolation and culture of neonatal rat cardiomyocytes

Neonatal rat cardiomyocytes (NRCM) were isolated from 2‐ to 3‐day‐old Sprague Dawley rat neonates using a protocol described previously (Mohamed et al., 2016). In brief, cardiomyocytes were obtained by digestion of neonatal rat hearts in ADS solution (116‐mM NaCl, 20‐mM HEPES, 1‐mM NaH_2_PO_4_, 5.5‐mM glucose, 5.5‐mM KCl 1‐mM MgSO_4_ ; pH 7.35) containing 0.6 mg·ml^−1^ collagenase A (Roche) and 0.6 mg·ml^−1^ pancreatin (Sigma). Cardiac fibroblasts were separated by plating on 10‐mm culture dishes for 1 hr to allow them to attach, leaving the myocytes in the media. Cardiomyocytes were plated on BD Falcon Primaria tissue culture plates in plating medium (68% DMEM, 17% M199, 10% horse serum, 5% FBS, and 2.5 μg·ml^−1^ amphotericin B) containing 5‐bromo‐2‐deoxyuridine (BrdU; 1‐μM) and incubated for 24 hr at 37°C. The next day, cardiomyocytes were twice washed with PBS and maintained in a maintenance medium (80% DMEM and 20% M199, 1% FBS, 2.5 μg·ml^−1^ amphotericin B, and 1‐μM BrdU) at 37°C, before use in experiments.

### 2.2 YAP luciferase assay

YAP activity was determined using a luciferase reporter assay as described by Tian, Yu, Tomchick, Pan, and Luo (2010). We generated adenoviruses expressing Gal4‐TEAD and UAS‐luciferase constructs to enable efficient gene transfer to cardiomyocytes. The Gal4‐TEAD4 construct was a gift from Dr Kunliang Guan (Addgene plasmid # 24640; http://n2t.net/addgene:24640; RRID:Addgene_24640), whereas the pUAS‐luc2 was a gift from Dr Liqun Luo (Addgene plasmid # 24343; http://n2t.net/addgene:24343; RRID:Addgene_24343; Potter, Tasic, Russler, Liang, & Luo, 2010). NRCM were seeded onto 24‐well plates. The following day, NRCM were treated with XMU‐MP‐1 (1, 3, and 5 μM) or DMSO. After 24 hr of incubation, cells were lysed, and the luciferase signal was measured using luciferase detection reagent (Promega). Data were normalised against the mean value of the control group.

### 2.3 YAP nuclear translocation

To assess YAP nuclear translocation, we used GFP‐YAP constructs to detect YAP sub‐cellular localisation in NRCM. We generated adenoviruses expressing GFP‐YAP to enable efficient gene transfer to NRCM. The GFP‐YAP construct (pEGFP‐C3‐hYAP1) was a gift from Dr Marius Sudol (Addgene plasmid # 17843; http://n2t.net/addgene:17843; RRID:Addgene_17843; Basu, Totty, Irwin, Sudol, & Downward, 2003). NRCM were seeded onto laminin‐coated coverslips in 24‐well plates. After overnight culture, the cells were treated with XMU‐MP‐1 (1–5 μM) for 24 hr. GFP signals were detected using fluorescent microscopy.

### 2.4 Cellular hypertrophy experiment

We analysed cardiomyocyte cell circumference and measured expression of the hypertrophic marker, brain natriuretic peptide (BNP), using a BNP‐luciferase reporter in cardiomyocytes. The day before the experiments, NRCM were cultured on laminin‐coated coverslips in a maintenance medium with 1% serum. They were then treated with phenylephrine (Sigma) at a dose of 50 μM for 72 hr with or without the addition of XMU‐MP‐1 at 1–5 μM. To determine cardiomyocyte size, cells were stained with an anti‐α‐actinin antibody (Sigma). The cell size (cell circumference) was then measured using ImageJ software. These experiments were carried out using NRCM isolated from six separate litters of rat neonates, to give *N* = 6 independent biological replications. At least *n* = 50 cardiomyocytes were analysed per treatment group in each experiment. To minimise variability due to different conditions between independent experiments, we normalised all cell size values against the mean value of the control group. Data are presented as fold induction relative to the mean value of the control group.

### 2.5 Apoptosis and cell viability assays

For the apoptosis assay, NRCM were seeded onto laminin‐coated coverslips in 24‐well plates. To induce apoptosis, NRCM were treated with 200‐μM hydrogen peroxide (Sigma) for 4 hr in the presence of XMU‐MP‐1 (1–5 μM) or DMSO as control. Apoptosis was detected using TUNEL assay (In situ Cell Death Detection Kit, Roche) following the protocol recommended by the manufacturer.

For cell viability assessment, we used MTT (thiazolyl blue tetrazolium bromide, Sigma) to stain viable cardiomyocytes following treatment with H_2_O_2_ (200 μM for 4 hr) with or without XMU‐MP‐1. The precipitated crystal dye was dissolved using solubilisation solution (0.1‐N HCl in isopropanol) before measurement of absorbance at 570 nm using a Multiskan Ascent Plate Reader. Data were normalised against the mean value of the control group.

### 2.6 In vivo experiments

Animal experiments were performed in accordance with the United Kingdom Animals (Scientific Procedures) Act 1986 and were approved by the University of Manchester Ethics Committee. Animal experiments are reported in compliance with the ARRIVE guidelines (Kilkenny et al., 2010; McGrath & Lilley, 2015) and with the recommendations made by the *British Journal of Pharmacology*. This study does not have any implications for replacement, refinement, and reduction.

We used wild type male C57Bl/6 mice (purchased from Envigo, UK) at 8 weeks of age with average weight of 27.7 ± 1.9 g at the start of the experiments. Mice were housed in the University of Manchester BSF Facility in a standard housing condition for laboratory animals. Mice were maintained in cages with a maximum of four mice per cage. They were kept in Techniplast GM 500 mouse cages, with a floor area of 500 cm^2^ and bedded with Aspen chip supplied by Datesand. Mice were maintained on a 12‐hr light/dark cycle in a controlled temperature of 19–22°C and humidity of 40–65% for 1 week before the experiments were started. Mice were fed with a standard chow diet (BK001, Special Diet Services, UK).

To produce a model of cardiac pressure overload, mice were subjected to transverse aortic constriction (TAC) or a sham operation (control). Mice were randomly grouped to either sham surgery (control) group or TAC group with or without XMU‐MP‐1 treatment. Before surgery, mice were anaesthetised with isoflurane (4–5% ) at a rate of 1 L·min^−1^ inhalation during induction and maintained at a dose of 2–3% at a rate of 1 L·min^−1^ during surgery. Mice were given analgesia immediately preceding surgery with buprenorphine (0.1 mg·kg^−1^ intraperitoneally). Details of the TAC procedure are described in our previous publications (Mohamed et al., 2016; Zi et al., 2014). In brief, the thorax cavity was opened to expose the aortic arch. By using a binocular stereomicroscope (Olympus), the TAC was carried out by tying a 7‐0 silk suture around a 27‐G needle overlying the aortic arch. The constriction site was located between the brachiocephalic trunk and the left common carotid artery. In our laboratory, this will normally produce a ~25–30 mmHg pressure gradient between the left carotid artery and the right carotid artery. For the sham group, the aortic arch was exposed as above, and a suture was passed around the back of the aorta without tying. Finally, the chest was sutured, and the mice were placed in a 30°C incubator for recovery before being returned to normal housing. We observed the mouse grimace scale every 2 hr after surgery up to 6 hr and then followed up the next day. A second dose of buprenorphine (0.1 mg·kg^−1^) was given if signs of pain were observed in the mice.

We performed TAC surgery in a total of 32 mice. Twelve mice were lost at day 0 and day 1 due to surgical complications. Twenty mice survived up to 3 weeks post‐TAC surgery. These animals were allocated to vehicle‐ or XMU‐MP‐1‐treated groups using a randomised block design as described by Festing, Overend, Das, Borja, and Berdoy (2004). Each cage was used as a blocking factor. Mice in the same cage were randomly assigned to two groups (TAC‐vehicle and TAC‐XMU‐MP‐1) using labelled paper.

XMU‐MP‐1 was dissolved in DMSO and injected intraperitoneally at a dose of 1 mg·kg^−1^ BW, given every 2 days. The dosing regimen was based on the pharmacokinetics data of XMU‐MP‐1 in a rodent model (Fan et al., 2016) and functional assays showing the effects of XMU‐MP‐1 injection (1 mg·kg^−1^) in reducing MOB1 phosphorylation in mice (Fan et al., 2016; Liu et al., 2018). XMU‐MP‐1 treatment started at 21 days post‐surgery for further 10 days. Control mice were injected with equal volumes of vehicle (DMSO). At the end of experiments, mice were killed by cervical dislocation, and the hearts were collected for further analysis. The subsequent analyses (echocardiography and heart weight/tibia length [HW/TL]) were performed in a blinded fashion (without knowledge of the treatments) for all of the groups.

### 2.7 Echocardiography analysis

Echocardiography was performed using a Visualsonics Vevo770 ultrasound machine fitted with a 14‐MHz transducer. Mice were anaesthesised with 1.5% isofluorane for this experiment. Details of echocardiography analysis were described in previous publications (Mohamed et al., 2016; Zi et al., 2014). In brief, mouse hearts were visualised in the two‐dimensional short‐axis view. Subsequently, M‐mode echocardiography was recorded to assess left ventricular end‐diastolic diameter (LVEDD), left ventricular end‐systolic diameter (LVESD), posterior wall (dPW), and interventricular septal (dIVS) thicknesses in diastole. Measurements were taken using the leading‐edge method for a minimum period of three cardiac beating cycles. The researcher conducting the analysis was blinded to mouse genotype and treatment. LV mass was calculated using the following formula: 1.055 × [(LVEDD + dPW + dIVS)^3^ − LVEDD^3^]. Whereas ejection fraction was calculated using following formula:

$$\mathrm{EF}=\frac{\left(\text{diastolic volume}-\text{systolic volume}\right)}{\text{diastolic volume}}*100.$$

### 2.8 Histological analysis and immunofluorescence

Heart tissues collected at the end of the experiment were cut into three parts; the apex and base parts were divided into two for protein and RNA analyses, whilst the middle part was used for histological study. The heart, liver, and kidney tissues were fixed using 4% paraformaldehyde in PBS for 24 hr. The following day, tissues were processed overnight using a Leica automated tissue processor and then were embedded in paraffin wax. The histological sections were prepared at 5‐μM thickness using a rotary microtome (Leica 2255). Masson's trichrome staining was performed for detection of fibrosis. Haematoxylin and eosin staining was performed for cardiomyocyte size measurement. Both analyses were performed using Panoramic 3D viewer software. Histological sections were also used for TUNEL assay (using a similar protocol described above) and for detection of Ki‐67 expression by immunofluorescence.

### 2.9 qRT‐PCR

RNA was extracted from the heart tissues using TRIzol. qRT‐PCR was conducted to measure the level of gene expression using SYBR Green (Agilent Technologies) or TaqMan (Applied Biosystems) reagents. Gene expression was quantified using the comparative C_T_ method (ΔΔC_T_ method) where the C_T_ value of the genes of interest were normalised to the C_T_ value of GAPDH gene expression.

### 2.10 Western blot

Protein was extracted from the heart tissues or NRCM using RIPA buffer. Protein level was measured using a BCA protein kit (Invitrogen). Western blot analysis to detect protein expression was performed using standard methods as described previously (Mohamed et al., 2016). The immuno‐related procedures used comply with the recommendations made by the *British Journal of Pharmacology*.

### 2.11 Data and statistical analysis

The data and statistical analysis comply with the recommendations of the *British Journal of Pharmacology* on experimental design and analysis in pharmacology (Curtis et al., 2018). Data are presented as mean ± SEM. Statistical analysis was performed using GraphPad Prism v.7.03. One‐way ANOVA was used to compare significance among groups. If ANOVA produced a significant value of *F* (*P* < .05) and there was no significant variance inhomogeneity, then the post hoc multicomparison analysis (Tukey's) was applied. For comparison between two groups, Student's *t* test was used. A *P* value of <.05 was considered significant. Echocardiography data including ejection fraction, LV end‐diastolic dimension and LV systolic dimension, as well as body weights , were analysed using two way ANOVA followed by post hoc multicomparison analysis (Tukey's). Non‐parametric test (Kruskal–Wallis test followed by Dunn's multiple comparison) was used to analyse band density data of phosphorylated/total MOB1.

Sample size for the in vivo experiments was estimated using power calculation analysis. Based on our previous study using Mst2 knockout mice in our laboratory (Zi et al., 2014), we predicted a 30% change in the HW/TL ratio between experimental groups with SD of 20% of the mean. Power calculations suggested that to detect a 30% change at a power of 80% with *α* = .05, a minimum of *N* = 7 mice per group were required. As we expected more variability and possible death after surgery, we decided for *n* = 10 in each of the TAC‐operated group (with or without XMU‐MP‐1 treatment).

### 2.12 Materials

XMU‐MP‐1 (Fan et al., 2016) was obtained from MedChem Express. Phenylephrine was obtained from Sigma (P6126). Antibodies used in this study include mouse anti‐α‐actinin (Sigma, cat no A7811), rabbit anti‐phospho‐MOB1 (Cell Signalling Technology, #8699S), rabbit anti‐MOB1 (Cell Signalling Technology, #13730S), rabbit anti‐active‐YAP (Cell Signalling Technology, #29495S), anti‐β‐actin HRP conjugate (Cell Signalling Technology, #122625), and anti‐Ki67 (Abcam, ab15580). Secondary antibodies used are Alexa Fluor® 647 anti‐mouse IgG (Jackson ImmunoReasearch Laboratories, Inc., 115‐605‐003), Alexa Fluor® 488 anti‐rabbit IgG (Jackson ImmunoReasearch Laboratories, Inc., 111‐545‐144), and anti‐rabbit IgG (Cell Signalling Technology, #7074S).

### 2.13 Nomenclature of targets and ligands

Key protein targets and ligands in this article are hyperlinked to corresponding entries in http://www.guidetopharmacology.org, the common portal for data from the IUPHAR/BPS Guide to PHARMACOLOGY (Harding et al., 2018), and are permanently archived in the Concise Guide to PHARMACOLOGY 2017/18 (Alexander, Fabbro et al., 2017; Alexander, Kelly et al., 2017).

## 3 RESULTS

### 3.1 Inhibition of Mst1 and Mst2 induces YAP activity in isolated cardiomyocytes

**Figure 1:**
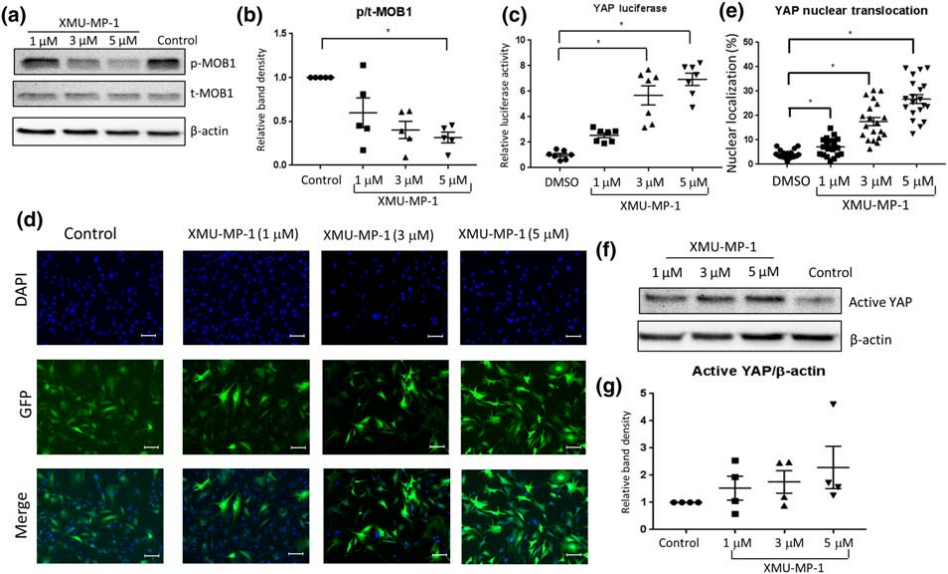
Treatment with XMU‐MP‐1 increases YAP activity in cultured neonatal rat cardiomyocytes (NRCM). (a) Representative western blots to show the reduction of MOB1 phosphorylation in NRCM following treatment with increasing doses of XMU‐MP‐1 (1–5 μM) for 24 hr in comparison with DMSO‐treated NRCM as control. (b) Quantification of band density of phosphorylated/total MOB1 expression level in NRCM following treatment with XMU‐MP‐1 (*N* = 5). (c) YAP activity was detected using YAP luciferase reporter system. XMU‐MP‐1 treatment (1–5 μM for 24 hr) significantly enhanced YAP luciferase activity in NRCM (*N* = 7). (d) Images showing the detection of YAP sub‐cellular localisation using GFP‐YAP construct (scale bar = 100 μm). (e) Quantification of the number of cells with nuclear YAP revealed that XMU‐MP‐1 treatment induced YAP nuclear translocation in cardiomyocytes (*N* = 20). (f) Western blot analysis and (g) quantification of band density to detect non‐phosphorylated YAP (active YAP) in NRCM treated with XMU‐MP‐1 (*N* = 4). Data shown in b, c, e, g are individual values with means ±SEM. ^*^ *P* < .05; significantly different as indicated; in b, non‐parametric Kruskal‐Wallis test with Dunn's multiple comparisons test, in c,and d, one‐way ANOVA with Tukey's multiple comparisons test

In this study, we used the recently identified inhibitor of Mst1/2, XMU‐MP‐1, to study the effects of pharmacological inhibition of these kinases in cardiomyocytes. To confirm that XMU‐MP‐1 treatment inhibited Mst1/2 activity in cultured NRCM, we analysed the phosphorylation of their substrate MOB1. Western blot analysis quantification of band density as presented in Figure 1a,b showed that XMU‐MP1 markedly reduced MOB1 phosphorylation in NRCM suggesting that this inhibitor was effective in suppressing Mst1/2 activity in cardiomyocytes.

Mst1 and Mst2 are the core components of the Hippo pathway that if activated will inhibit the co‐transcriptional activity of downstream effector YAP via Lats1/2. To investigate if Mst1/2 inhibition in cardiomyocytes induces YAP activity, we used the luciferase reporter system to monitor YAP activity. We inserted the components of the reporter system (the GAL4‐TEAD and UAS‐Luc constructs) into adenoviral vectors to achieve >95% transfection efficiency in NRCM. In this system, XMU‐MP‐1 significantly increased YAP activity by >5‐fold at doses of 1–5 μM (Figure 1c). Data were presented as fold increase relative to control to reduce variation between batches of experiments. As YAP activity is associated with its nuclear translocation, we then monitored YAP sub‐cellular localisation using a GFP‐YAP construct. Consistent with the data from the luciferase reporter assay, YAP nuclear translocation was increased in NRCM treated with XMU‐MP‐1 (Figure 1d,e). In agreement, western blot analysis also indicated an increase of active YAP expression in NRCM following XMU‐MP‐1 treatment (Figure 1f,g).

### 3.2 Inhibition of Mst1/2 attenuates phenylephrine‐induced hypertrophy in cardiomyocytes

**Figure 2:**
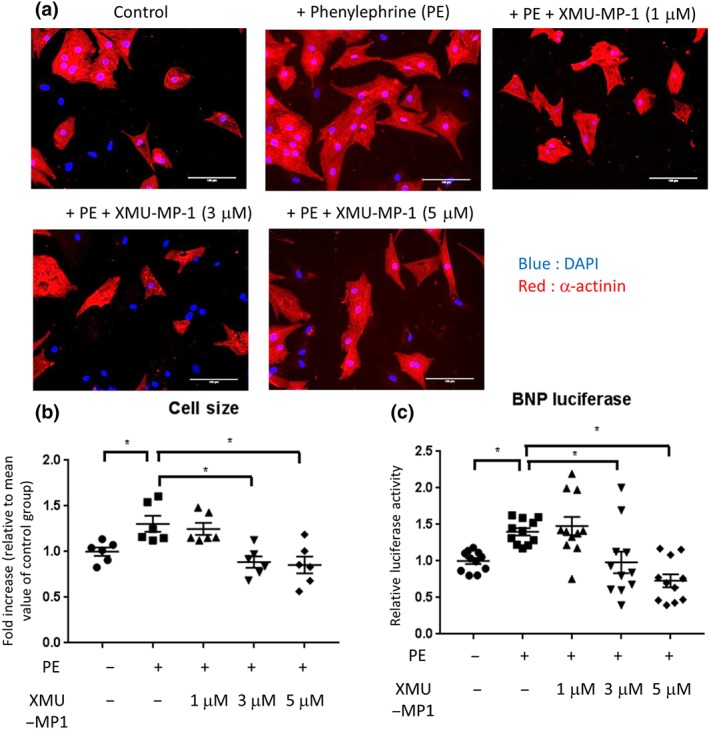
Treatment with Mst1/2 inhibitor XMU‐MP‐1 ameliorates cardiomyocyte hypertrophy in response to phenylephrine (PE) stimulation. (a) Representative images of NRCM stained with DAPI (blue) and α‐actinin (red). NRCM were stimulated with 50‐μM PE for 72 hr in the presence of XMU‐MP‐1 (1–5 μM) or DMSO as control. Cardiomyocyte size was determined using ImageJ software. (b) Quantification of cardiomyocytes size suggested that NRCM treated with XMU‐MP‐1 at doses of 3 and 5 μM displayed significantly less hypertrophic response than control cells. *N* = 6 independent experiments with 50+ cells analysed per group in each independent experiment. (c) Expression of hypertrophy marker BNP was examined by using BNP‐luciferase reporter system. In agreement with cell size measurement, XMU‐MP‐1 treatment at 3–5 μM significantly reduced BNP‐luciferase expression. *N* = 11; scale bar = 100 μm. Data shown in b and c are individual values with means ±SEM. ^*^ *P* < .05; significantly different as indicated; one‐way ANOVA with Tukey's multiple comparisons test

We and others have shown that both Mst1 and Mst2 are involved in regulating cardiomyocyte hypertrophy (Del Re et al., 2010; Zi et al., 2014). Therefore, to investigate whether XMU‐MP‐1 affects the hypertrophic response, we treated NRCM with phenylephrine (50 μM) for 3 days with or without the addition of XMU‐MP‐1 (1–5 μM). As indicated by cell size measurement, the hypertrophic response was attenuated following XMU‐MP‐1 treatment (3 and 5 μM), whereas the lowest concentration (1 μM) was ineffective (Figure 2a,b). However, there was no difference in cell size between NRCM treated with 3‐ and 5‐μM XMU‐MP‐1, suggesting that the treatment reached its maximum effect at 3 μM. To further confirm our finding, we used an adenovirus mediated luciferase reporter system to monitor the expression of the hypertrophic marker, BNP. The data from the BNP‐luciferase system were consistent with the findings from the cell size measurement (Figure 2c). Both the cell size measurement and BNP‐luciferase data are presented as fold increase relative to control to reduce variation between batches of experiments.

### 3.3 Mst1/2 inhibition reduces apoptosis and cell death following stress

**Figure 3:**
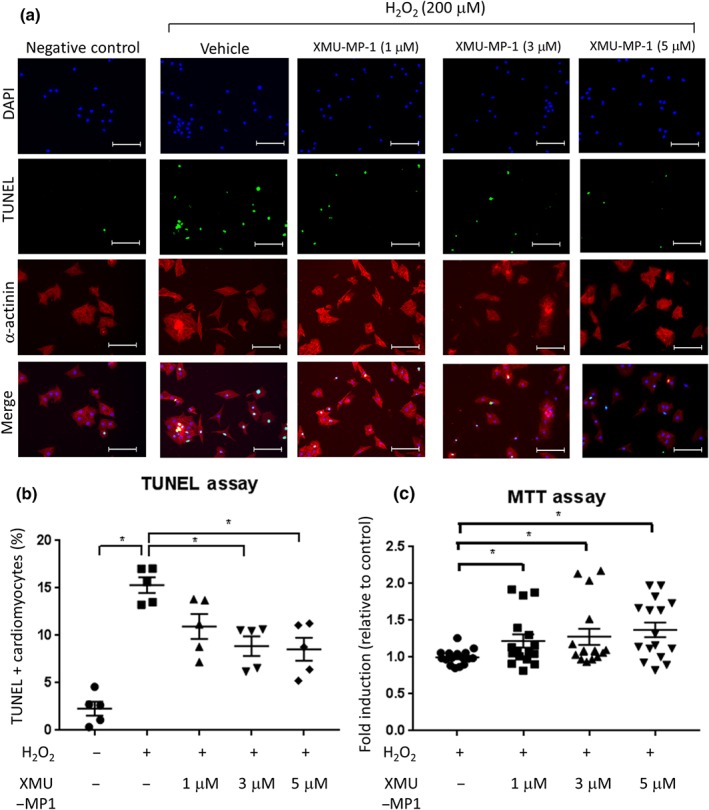
Effects of Mst1/2 inhibitor on apoptosis and cell survival in cultured NRCM. (a) Representative images of TUNEL assay to detect apoptosis in NRCM. Cells were treated with 200‐μM H_2_O_2_ for 4 hr to induce oxidative stress. XMU‐MP‐1 or DMSO (control) was added at the same time as H_2_O_2_. NRCM were stained with α‐actinin (red), TUNEL (green), and DAPI (blue). *N* = 5, scale bar = 100 μm. (b) Quantification of TUNEL positive cells revealed that NRCM treated with XMU‐MP‐1 (3 and 5 μM) displayed significantly lower level of apoptosis (*N* = 5). (c) MTT assay was conducted to assess the level of cell survival following stimulation with 200‐μM H_2_O_2_ for 4 hr. Results indicated that XMU‐MP‐1 treatment increased NRCM survival from H_2_O_2_ stimulation. *N* = 16. Data shown in b and c are individual values with means ±SEM. ^*^ *P* < .05; significantly different as indicated; one‐way ANOVA with Tukey's multiple comparisons test

Mst1 and Mst2 exert pro‐apoptotic activity in a number of different cell types (Galan & Avruch, 2016). To investigate whether XMU‐MP‐1 treatment inhibits apoptosis in cardiomyocytes, we treated NRCM with H_2_O_2_ (200 μM) for 4 hr to induce oxidative stress and eventually apoptosis, which was assessed by TUNEL assay. As shown in Figure 3a,b, addition of XMU‐MP‐1 (1–5 μM ) significantly reduced the number of TUNEL positive cells. In agreement with the inhibition of the hypertrophic response, XMU‐MP‐1 was effective in cells treated with the higher concentrations (3‐ and 5‐μM). As in the hypertrophy experiment, there was no difference between NRCM treated with 3‐ and 5‐μM XMU‐MP‐1, indicating that the maximum effect in reducing apoptosis was reached at 3 μM. To confirm this finding, we performed MTT assays to measure cell survival. We found that treatment with XMU‐MP‐1 significantly increased cell survival by 25–30% (Figure 3c). Together, our findings indicated that XMU‐MP‐1 treatment protected cardiomyocytes from apoptotic cell death and increased cell survival following oxidative stress.

### 3.4 Mst1/2 inhibition increases neonatal cardiomyocyte proliferation

**Figure 4:**
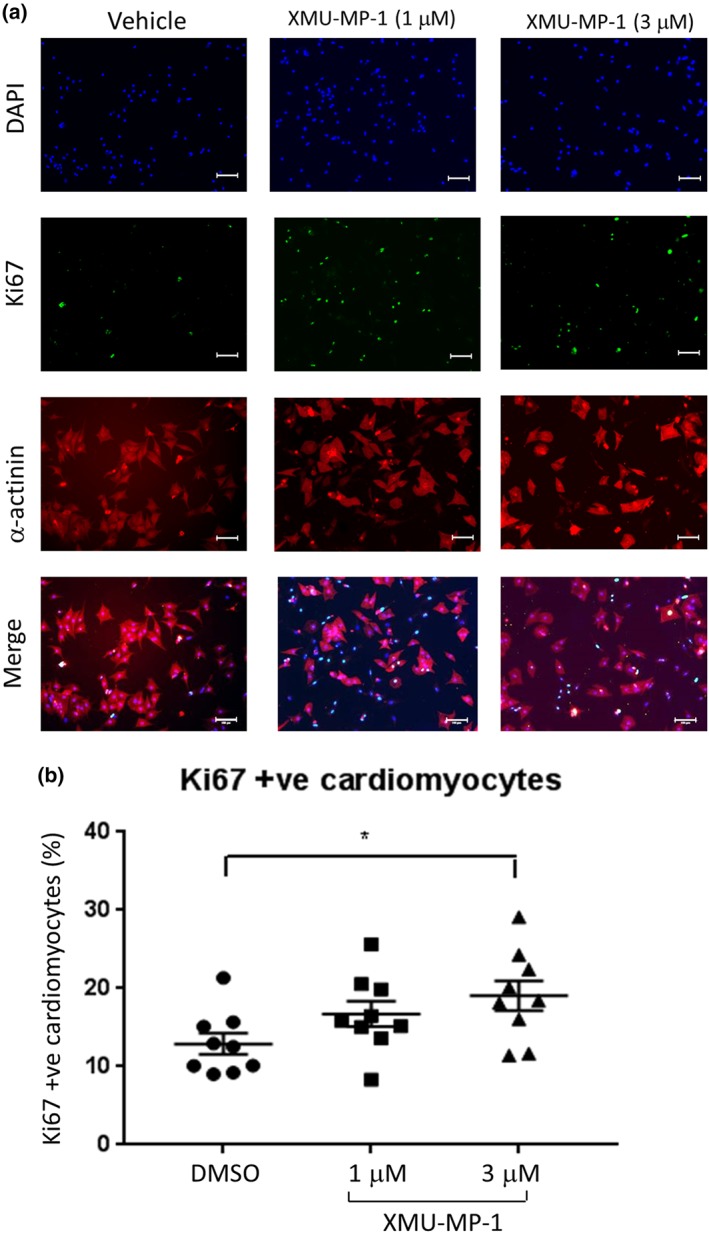
Mst1/2 inhibition using XMU‐MP‐1 increases NRCM proliferation. (a) Representative images of NRCM stained with α‐actinin (red), Ki‐67 (green), and DAPI (blue). NRCM were treated with XMU‐MP‐1 (1–3 μM) for 24 hr prior to fixation and immunostaining. (b) Analysis of Ki‐67 positive cells indicated that treatment with XMU‐MP‐1 at a dose of 3 μM significantly increased the number cardiomyocytes expressing Ki‐67. *N* = 9; scale bar = 100 μm. Data shown are individual values with means ±SEM. ^*^ *P* < .05; one‐way ANOVA with Tukey's multiple comparisons test

Inhibition of the Hippo pathway and activation of YAP are known to induce cell proliferation (Heallen et al., 2013; Leach et al., 2017; Lin et al., 2014). To examine if XMU‐MP‐1 stimulated proliferation of cultured NRCM, we assessed the level of Ki67 expression, which is known as a cell proliferation marker. As previous experiments suggested that the maximum effect of XMU‐MP‐1 treatment was observed at 3‐μM, in this experiment, we treated NRCM with 1‐ and 3‐μM XMU‐MP‐1. Data presented in Figure 4a,b showed that following XMU‐MP‐1 treatment at 3 μM, NRCM displayed a slight but significant increase in the number of Ki67 positive nuclei.

### 3.5 Treatment with Mst1/2 inhibitor improves cardiac function following pressure overload

**Figure 5:**
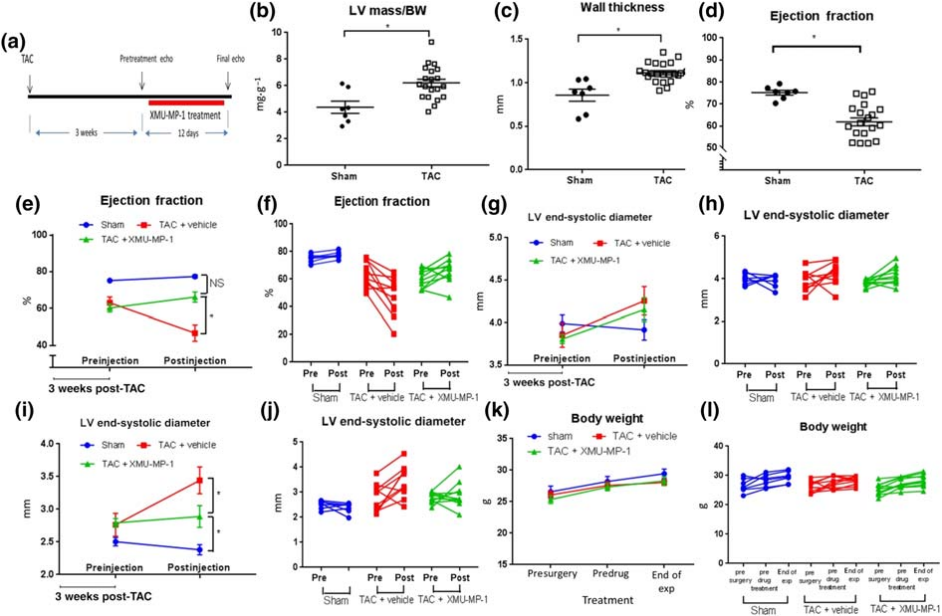
Treatment with XMU‐MP‐1 improves cardiac function and reduces LV dilatation following transverse aortic constriction (TAC) in mice. (a) Schematic diagram of the experimental procedure. Wild type (C57Bl/6) mice were subjected to TAC to induce pressure‐overload hypertrophy. After 3 weeks of TAC, mice were either treated with XMU‐MP‐1 (1 mg·kg^−1^ body weight per every alternate day) or DMSO as control. Echocardiography analysis was conducted before the first treatment and at day 12 after the first injection. (b) Echocardiography analysis at 3 weeks after TAC (before treatment with XMU‐MP‐1 or vehicle) showed that the TAC procedure markedly increased cardiac size as indicated by (b) LV mass/body weight measurement and (c) wall thickness. Sham, *N* = 7; TAC, *N* = 20. (d) Cardiac function (ejection fraction) was significantly reduced in TAC‐treated mice at 3‐week time point. (e) Treatment with XMU‐MP‐1 preserved cardiac function from further deterioration as shown in the vehicle‐treated mice. Dot plot of mean value of ejection fraction (e) and grouped column before–after scatter plot (f) showed that at the end of the experiment, the XMU‐MP‐1‐treated mice displayed significantly better cardiac function compared to vehicle‐treated mice. (g) There was no significant difference in LV end‐diastolic dimension between XMU‐MP‐1 and vehicle group as shown by (g) mean value dot plot and (h) before–after scatter plot. (i) However, the LV end‐systolic dimension was significantly smaller in XMU‐MP‐1‐treated mice compared to vehicle‐treated mice. (j) Grouped column before–after scatter plot of LV end‐systolic diameter. (k) Body weight was not significantly changed following surgery and drug treatment as shown in the (k) dot plot of mean values and (l) before–after scatter plot. Sham, *N* = 7; vehicle‐treated mice, *N* = 10; XMU‐MP‐1‐treated mice, *N* = 10. Data shown in B, C and D are individual values with means ± SEM. Data in panels E, G, I and K show mean ± SEM whereas data shown in F, H, J and L are the original individual values. ^*^ *P* < .05; significantly different as indicated; .in b, c, d, unpaired Student's t‐test; in e, g, I, k, two way ANOVA with Tukey's multiple comparisons test

In vitro experiments using cultured neonatal cardiomyocytes clearly showed that XMU‐MP‐1 treatment reduced phenylephrine‐induced hypertrophy and inhibited apoptosis following oxidative stress. Therefore, we hypothesised that XMU‐MP‐1 treatment would be beneficial to control cardiac remodelling in vivo. To address this question, we subjected wild type mice (C57Bl/6) to TAC for 3 weeks to induce cardiac hypertrophy by pressure overload and then treated the mice with XMU‐MP‐1 or vehicle (DMSO) once the hypertrophy was established (Figure 5a). Echocardiography assessment indicated that after 3 weeks, the TAC‐treated mice showed signs of substantial cardiac hypertrophy and reduced cardiac function, as indicated by LV mass/body weight ratio, wall thickness, and ejection fraction, compared to sham‐operated mice (Figure 5b–d). We then treated TAC‐stimulated mice with either a low dose of XMU‐MP‐1 (1 mg·kg^−1^, given every 2 days) or vehicle (DMSO, equal volume) and assessed cardiac phenotype 12 days after the first treatment. We found that XMU‐MP‐1‐treated mice exhibited significant improvement of cardiac function as indicated by ejection fraction, compared with vehicle‐treated TAC mice (Figure 5e,f). Equally important, XMU‐MP‐1 treatment protected mice from excessive LV dilatation as indicated by LV end‐systolic dimension compared with vehicle‐treated TAC mice although the LV end‐diastolic dimension was not different (Figure 5g–j). It is important to note that the body weights of the mice were not significantly changed following TAC or sham surgery or after treatment with XMU‐MP‐1 or vehicle (Figure 5k,l).

### 3.6 Effect of XMU‐MP‐1 treatment on cardiomyocyte size

**Figure 6:**
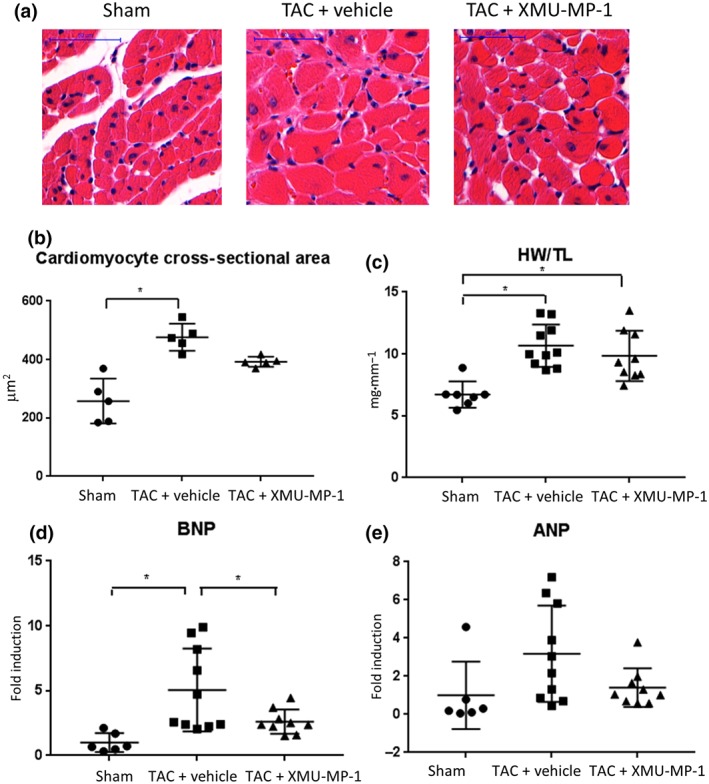
Reduction in cardiomyocyte size in TAC‐induced mice following treatment with Mst1/2 inhibitor. (a) Representative images of haematoxylin–eosin stained histological sections of the heart tissues from sham, TAC‐vehicle, and TAC‐XMU‐MP‐1 groups of mice. Scale bar = 50 μm. (b) Analysis of cardiomyocyte cross‐sectional area showed that there was a significant difference in cardiomyocyte size between the sham and TAC‐vehicle groups; however, there was no difference between the TAC mice treated with XMU‐MP‐1 compared with the sham group (sham, *N* = 5; TAC‐vehicle, *N* = 5; TAC‐XMU‐MP‐1, *N* = 5, with at least *n* = 100 cardiomyocytes analysed in each mouse). (c) In contrast, there was no significant difference in heart weight/tibia length ratio (HW/TL) between TAC‐vehicle and TAC‐XMU‐MP‐1 group. (d) On the other hand, the expression of hypertrophic markers BNP and ANP (e) indicated a significant reduction in BNP expression but not in ANP expression in TAC‐XMU‐MP‐1 group compared to TAC‐vehicle group (sham, *N* = 6; TAC‐vehicle, *N* = 10; TAC‐XMU‐MP‐1, *N* = 9). Data shown in b, c, d, e are individual values with means ±SEM. ^*^ *P* < .05; significantly different as indicated; one‐way ANOVA with Tukey's multiple comparisons test. ANP, atrial natriuretic peptide; BNP, brain natriuretic peptide

We next analysed the effect of XMU‐MP‐1 on hypertrophy, afterTAC. We analysed histological sections of the cardiac tissues focusing on measuring the cardiomyocyte cross‐sectional area. We observed a significant increase in cardiomyocytes size in the TAC + vehicle group, compared with the sham group. In contrast, there was no significant difference in cardiomyocyte size between the sham group and TAC mice treated with XMU‐MP‐1 (Figure 6a,b), indicating that XMU‐MP‐1 treatment reversed the cellular hypertrophy response in vivo. However, we did not observe any significant difference in HW/TL ratio between mice treated with XMU‐MP‐1 and controls (Figure 6c). In support of the cell size data, qRT‐PCR analysis revealed a significant reduction in the expression of hypertrophic marker BNP in XMU‐MP‐1 treated TAC mice (Figure 6d). On the other hand, expression of atrial natriuretic peptide (ANP) was not statistically different between groups, although the mean value was higher in the TAC + vehicle mice, compared with in mice significant the other groups.

### 3.7 Mst1/2 inhibitor reduces apoptosis and fibrosis in TAC‐induced hypertrophy

**Figure 7:**
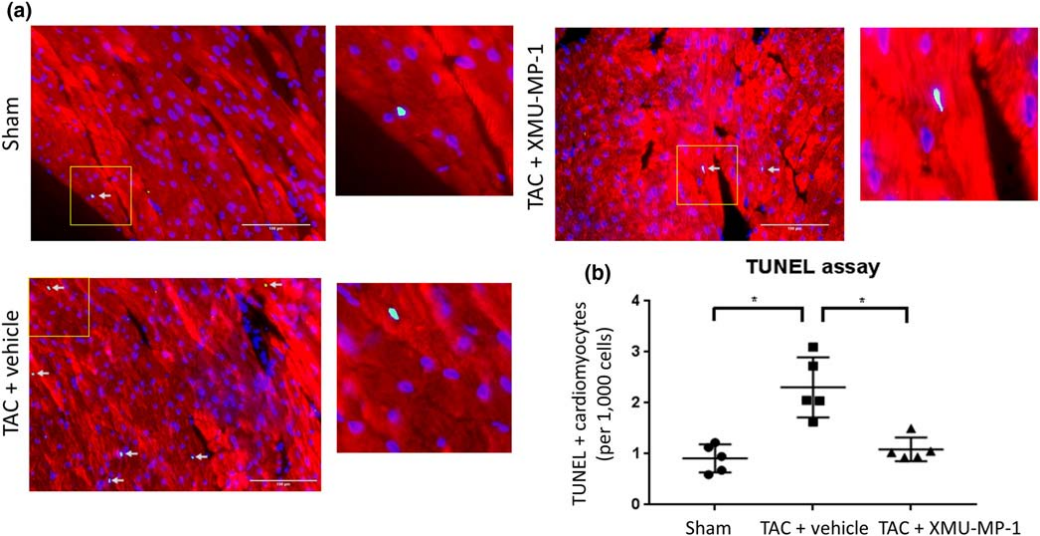
Apoptosis level is reduced in TAC mice treated with XMU‐MP‐1. (a) Representative images of TUNEL staining in cardiac histological sections (red = α‐actinin, green = TUNEL, blue = DAPI, scale bar = 100 μm). (b) Quantification of TUNEL positive cardiomyocytes revealed a significant reduction of apoptosis in XMU‐MP‐1 group compared to vehicle control (sham, *N* = 5; TAC‐vehicle, *N* = 5; TAC‐XMU‐MP‐1, *N* = 5). Data shown are individual values with means ±SEM. ^*^ *P* < .05; significantly different as indicated; one‐way ANOVA with Tukey's multiple comparisons test

In vitro evidence strongly suggested that Mst1/2 inhibition reduced cardiomyocyte apoptosis under oxidative stress conditions. Therefore, we performed TUNEL analysis in the cardiac tissue sections of XMU‐MP‐1‐treated mice and controls. Results shown in Figure 7a,b suggest that XMU‐MP‐1 significantly reduced the apoptosis level in the heart of TAC mice. These data are in line with the previous reports using mice with genetic knockout of Mst1 and Mst2 (Del Re et al., 2010; Zi et al., 2014).

**Figure 8:**
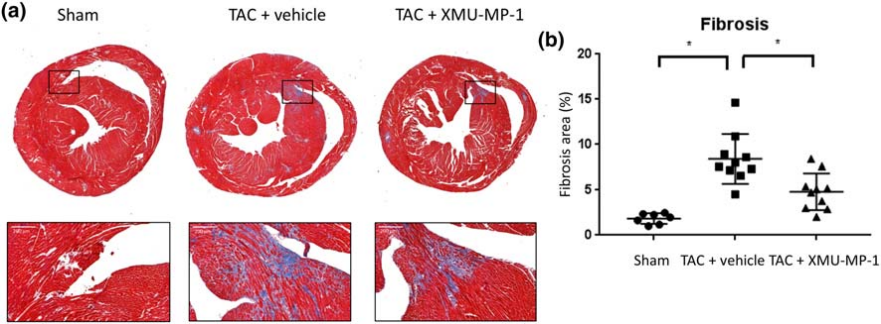
Fibrosis level is reduced in TAC mice treated with XMU‐MP‐1. (a) Representative images of cardiac histological sections stained with Masson's trichrome to detect fibrosis. Scale bar = 200 μm. (b) Level of fibrosis was assessed by measuring blue area relative to the whole cardiac section. Treatment with XMU‐MP‐1 significantly reduced fibrosis in TAC‐induced hypertrophy model. (sham, *N* = 7; TAC‐vehicle, *N* = 10; TAC‐XMU‐MP‐1, *N* = 10). Data shown are individual values with means ±SEM. ^*^ *P* < .05; significantly different as indicated; one‐way ANOVA with Tukey's multiple comparisons test

To examine the effect of Mst1/2 inhibition on the development of cardiac fibrosis, we analysed histological sections stained with Masson's trichrome. Consistent with our analyses on hypertrophy and apoptosis, we observed significant reduction in fibrosis in cardiac tissue from XMU‐MP1‐treated mice, compared with vehicle‐treated TAC mice (Figure 8a,b). Together with the apoptosis data, these may explain the underlying mechanism(s) of the beneficial effects of XMU‐MP‐1 treatment in improving cardiac function during pressure overload hypertrophy.

### 3.8 Adult cardiomyocyte proliferation is not altered after XMU‐MP‐1 treatment in vivo

**Figure 9:**
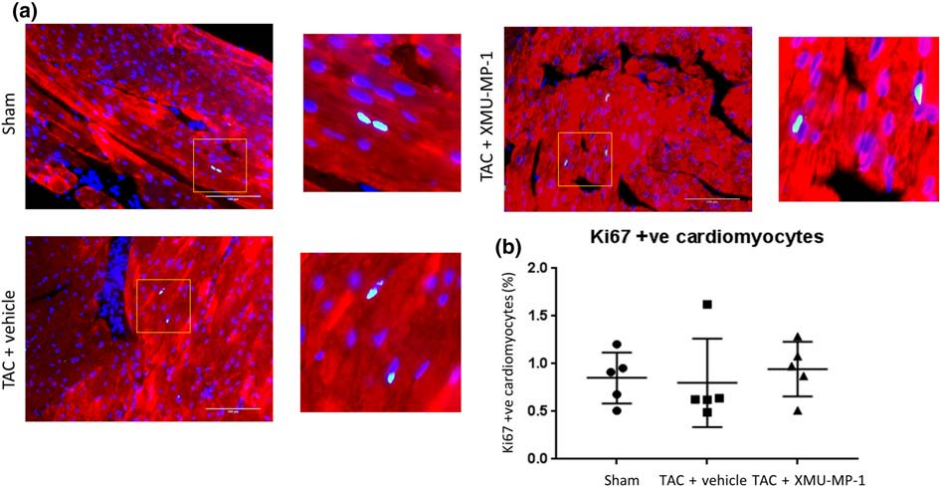
XMU‐MP‐1 treatment in vivo did not alter adult cardiomyocyte proliferation. (a) Representative images of immunofluorescence analysis to detect Ki67 expression in cardiac tissue sections of TAC‐ or sham‐treated mice. Scale bar = 100 μm. (b) The number of Ki67 positive myocytes did not differ between the three groups of mice suggesting that treatment with XMU‐MP‐1 did not alter adult cardiomyocytes proliferation rate in vivo (sham, *N* = 5; TAC‐vehicle, *N* = 5; TAC‐XMU‐MP‐1, *N* = 5). Data shown are individual values with means ±SEM

Our in vitro experiments showed that treatment with XMU‐MP‐1 at 3 μM increased proliferation of neonatal cardiomyocytes as indicated by Ki‐67 staining. To investigate if treatment with XMU‐MP‐1 affected the proliferation of adult cardiomyocyte in vivo, we performed immunofluorescence analysis of the Ki67 expression in the heart sections of TAC mice. We found no difference in the level of Ki67 positive cells between XMU‐MP‐1‐treated mice and controls (Figure 9a,b) indicating a different response to XMU‐MP‐1 treatment between neonatal and adult cardiomyocytes with regard to the regulation of cell proliferation.

### 3.9 XMU‐MP‐1 treatment does not cause significant abnormality in the liver or kidney

As the components of the Hippo pathway are expressed in many organs, it is important to assess the possible effects of Mst1/2 inhibitor treatment on major organs. As a first step to assess the effects of XMU‐MP‐1 in other organs, we examined the liver and kidney from mice treated with XMU‐MP‐1. Analysis of histological sections (Masson's trichrome staining) indicated that there was no significant change in the fibrosis level in either the liver or the kidney after XMU‐MP‐1 treatment (Figure S1A–D). Consistent with these findings, the levels of serum alanine amino transferase and creatinine kinase, as indicators of liver and kidney function, respectively, were not altered in mice treated with XMU‐MP‐1 (Figure S1E,F). These findings suggested that XMU‐MP‐1 treatment in vivo at the dose and duration used in our experiments was not likely to cause any abnormalities in the liver or kidney.

## 4 DISCUSSION

The key finding of this study is that treatment with the Mst1/2 inhibitor XMU‐MP‐1 improved cardiac function and reduced apoptosis and fibrosis in a mouse model of cardiac hypertrophy due to pressure overload. This provides the first evidence that pharmacological inhibition of key components of the Hippo pathway (Mst1/2) is beneficial in the heart under stress conditions.

Previous studies using a genetic approach have shown that inhibition of the Hippo pathway components, which eventually results in the activation of its main effector YAP, is beneficial in attenuating the adverse effects of pathological stimuli in the heart. For example, we have shown that genetic knockout of Mst2 produced a protective effect in the heart under pressure overload conditions. Mst2 knockout mice displayed less hypertrophy, fibrosis, and apoptosis following TAC stimulation (Zi et al., 2014). Likewise, inhibition of Mst1 by expressing a dominant negative mutant form of this kinase reduced apoptosis and protected against cardiac dysfunction following myocardial infarction (Odashima et al., 2007). In keeping with this finding, overexpression of Mst1 caused dilated cardiomyopathy due to excessive apoptosis (Yamamoto et al., 2003). Regulation of the pro‐apoptotic protein, https://www.guidetopharmacology.org/GRAC/ObjectDisplayForward?objectId=2845 has been suggested as a possible mechanism of Mst1‐dependent apoptosis in the cardiomyocytes (Del Re et al., 2014). In addition, activation of JNK and protein cleavage by caspase is also involved in Mst1‐mediated apoptosis (Ura, Masuyama, Graves, & Gotoh, 2001).

Other components of the Hippo signalling pathway also play important roles in the heart. Sav1 is an adaptor molecule that positively modulates Hippo signal activation (Misra & Irvine, 2018). Heallen et al. showed that ablation of Sav1 produced beneficial effects in the heart in a model of myocardial infarction (Heallen et al., 2013). It was suggested that the increase in cardiomyocyte proliferation was the underlying mechanism for the reduction of infarct size and preservation of cardiac function in Sav1 knockout mice (Heallen et al., 2013; Leach et al., 2017). Lats1/2, the other members of the pathway which form the central kinase cascade, also display key roles in the heart. Consistent with the observations in Mst1 and Mst2 mouse models, genetic inhibition of Lats2 using a dominant negative mutant attenuated apoptosis and improved the phenotype following myocardial infarction in mice (Matsui et al., 2008).

The main downstream effector of the Hippo signalling pathway is the transcriptional activator YAP. Hippo signalling deactivates YAP by phosphorylation and cytoplasmic retention (Misra & Irvine, 2018). Thus, the premise is that if inhibition of the Hippo signal is beneficial in the heart, then activation of YAP must be beneficial as well. Observations on transgenic and knockout models of YAP strongly support this notion. Expression of constitutively active YAP increased cardiomyocyte proliferation, reduced infarct size, and improved cardiac function in a model of myocardial infarction (Lin et al., 2014; Xin et al., 2013). In contrast, mice with cardiomyocyte specific knockout of YAP exhibited apoptosis, hypertrophy, LV dilatation, fibrosis, and reduction of contractility. Moreover, in response to myocardial infarction, YAP‐deficient mice displayed more severe injury and reduction of cardiac function (Del Re et al., 2013; Xin et al., 2013). Taken together, there is strong evidence from the genetic experiments that inhibition of the Hippo pathway and/or induction of YAP activity in the heart is beneficial. However, despite the strong evidence from the genetic models, little is known if pharmacological inhibition of the Hippo pathway will also produce beneficial effects in the heart.

Although the Hippo pathway has been relatively well characterised, there are only a limited number of low MW compounds that are known to positively or negatively modulate this pathway. Pharmacological modulation of the Hippo pathway is difficult to achieve because most of the upstream regulators of YAP do not have enzymic activity (Johnson & Halder, 2014). Of several signalling branches that can modulate YAP, only the Mst1/2–Lats1/2 axis consists principally of kinases, making this pathway an attractive target for inhibitor development (Johnson & Halder, 2014). Anand and co‐workers identified the first inhibitor of Mst1. They reported a small molecule (9E1) that has a high potency in inhibiting Mst1 (IC_50_ = 45 nM; Anand et al., 2009). Using a cellular model, they found effectivity in inhibiting Mst1 by measuring Histone H2B phosphorylation (Anand et al., 2009). However, the efficacy in an in vivo model has not been tested yet. The pan‐Mst1/2 inhibitor XMU‐MP1, which was identified from high throughput screening of a kinase inhibitor library, was first used for tissue repair and regeneration. In contrast to compound 9E1, this compound has been used in vivo to treat liver injury in mice, and the result was very promising (Fan et al., 2016). With IC_50_ values of 71 and 38 nM for Mst1 and Mst2 inhibition, respectively, XMU‐MP‐1 can be regarded as a highly potent inhibitor for these kinases. Fan et al. have also demonstrated the selectivity of XMU‐MP‐1 by performing analysis against a panel of 468 kinases. Aside from the MST kinases, only https://www.guidetopharmacology.org/GRAC/ObjectDisplayForward?objectId=1936 and https://www.guidetopharmacology.org/GRAC/ObjectDisplayForward?objectId=2156 displayed high affinity to XMU‐MP‐1, neither of which is known to regulate cardiac hypertrophy.

More recently, a compound, TT‐10 has been described that can induce YAP activity by directly enhancing the transcriptional activity of the YAP–TEAD complex, although the exact molecular mechanism is not completely described. This compound was able to induce cardiomyocyte proliferation both in vitro and in vivo, and treatment with TT‐10, albeit at very high dose, improved the cardiac phenotype in a mouse model of myocardial infarction (Hara et al., 2018).

Despite all the promising findings in the setting of myocardial infarction, little is known on the effect of pharmacological Hippo pathway suppression in response to pressure overload stress in the heart. Our data are the first to show the beneficial effects of Mst1/2 inhibition following TAC surgery in mice. Both the in vitro and in vivo experiments indicate that Mst1/2 inhibition using XMU‐MP‐1 improves hypertrophic remodelling and reduces apoptosis. More importantly, we found marked improvement of cardiac contractility of TAC‐induced mice after treatment with XMU‐MP‐1, possibly as a direct consequence of reduced apoptosis and fibrosis in the heart. However, despite reduction in cardiomyocyte size in mice treated with XMU‐MP1, there was no difference in the HW/TL ratio. This might be due to the reduction of apoptosis and hence lower cell death in XMU‐MP‐1‐treated mice. Thus, there might be a greater number of cardiomyocytes, and hence, the HW/TL ratio is comparable with that of control mice, despite smaller cardiomyocyte size.

One major aspect of drug development is their safety with respect to potential side effects. It is clear that Mst1/2 inhibition and YAP induction induces cell proliferation in vitro and in vivo. Previous studies using XMU‐MP‐1 (Fan et al., 2016) and the YAP activator TT‐10 (Hara et al., 2018) showed increases in proliferation of hepatocytes, small intestinal epithelial cells, and cardiomyocytes following in vivo treatment with the compounds. It is understandable that these data may raise some concerns with regard to the possible development of tumours or cancers, following prolonged use of these compounds. In this study, we tried to use a low dose of XMU‐MP1 (1 mg·kg^−1^ BW), which was given every 48 hr, to minimise possible side effects in other organs. Although our in vitro data using NRCM showed a slight effect of XMU‐MP‐1 in inducing proliferation of neonatal cardiomyocytes, our in vivo regimen did not stimulate adult cardiomyocyte proliferation, despite its effectiveness in preserving cardiac function and controlling apoptosis. We also found that this dose did not cause any significant damage in other major organs as indicated by liver and kidney function tests and histological analysis. Together, our data suggest that at this dose and duration of treatment, XMU‐MP‐1 is safe, while it is effective in protecting the heart from pressure overload damage in mice. However, the effects of longer term and higher doses remain to be determined.

Apart from its key role in the heart, the Hippo pathway also plays essential roles in different organs and cell types. The pro‐proliferative and anti‐apoptotic nature of Mst1/2 have been described to be important in pancreatic beta cells and hepatocytes. Inhibition of these kinases is beneficial to prevent pancreatic damage and development of diabetes as well as to induce liver regeneration (Ardestani et al., 2014; Fan et al., 2016). Mst1 inhibition also produces positive effects during adult somatic cell reprogramming to induced pluripotent stem cells (Robertson et al., 2017). However, total genetic ablation of Mst1 and Mst2 is also associated with the development of cancer in the liver and intestines (Song et al., 2010; Zhou et al., 2011). Thus, in the cancer field, it is the activation of the Hippo pathway (or inhibition of YAP) that would likely produce favourable effects. This also underlines the double‐edged sword effect of Hippo pathway inhibition. However, in the mouse models, it requires both Mst1 and Mst2 to be completely ablated during embryonic development to produce cancer in the adult. Therefore, a possible approach to minimise carcinogenic effects would be to develop specific inhibitors for each isoform (Mst1 or Mst2 only), alongside minimising the dose and the duration of treatment as we show in our study.

In a clinical setting, it is likely that long‐term treatment with an anti‐hypertrophic agent would be needed to prevent the development of heart failure as well as reversing the pathological hypertrophic growth. Thus, further study is required to test the effects of longer XMU‐MP‐1 treatment in a chronic model of cardiac pressure overload, while it would also be important to evaluate whether the protective effects remain following drug withdrawal. Although these experiments are beyond the scope of the current study, our study does provide the first proof of concept that inhibition of the core components of the Hippo signalling pathway (i.e., Mst1 and Mst2) using pharmacological compounds produces beneficial effects in the heart in the pathological setting.

In summary, our data add to a growing body of evidence that targeting the Hippo pathway for the treatment of heart disease will be a promising area to be developed in the future. In addition, the fact that most of the components of this pathway are expressed and differentially regulated in diseased human hearts (Leach et al., 2017; Zi et al., 2014) make this pathway an interesting target for therapy.

## CONFLICT OF INTEREST

The authors have no conflicts of interest to disclose.

## AUTHOR CONTRIBUTIONS

D.O. conceived the scientific idea, supervised the whole project, planned and designed the experiments, analysed and interpreted the data, and wrote the manuscript. E.T. planned, designed, and performed in vitro and in vivo experiments and analysed and interpreted the data. A.B.N., Y.S.C., and T.A.B. helped in in vitro experiments. M.Z. and S.P. performed the mouse surgery. N.S. helped in in vivo data analysis and reviewed and proofread the manuscript. E.J.C. supervised in vivo experiments. S.A. helped in designing the experiments and supervised the project.

## DECLARATION OF TRANSPARENCY AND SCIENTIFIC RIGOUR

This Declaration acknowledges that this paper adheres to the principles for transparent reporting and scientific rigour of preclinical research as stated in the *BJP* guidelines for https://bpspubs.onlinelibrary.wiley.com/doi/full/10.1111/bph.14207, https://bpspubs.onlinelibrary.wiley.com/doi/full/10.1111/bph.14208, and https://bpspubs.onlinelibrary.wiley.com/doi/full/10.1111/bph.14206, and as recommended by funding agencies, publishers and other organisations engaged with supporting research.

## Supporting information

Figure S1.
Analysis of XMU‐MP‐1 effects in liver and kidney **A)** Representative images of liver tissue sections stained with Masson's trichrome and **B)** Quantification of liver fibrosis showed that there is no significant difference in liver fibrosis between vehicle treated and XMU‐MP‐1 treated mice (vehicle, N= 5; XMU‐MP‐1, N= 5). **C)** Images of kidney histological sections stained with Masson's trichrome. **D)** Assessment of fibrosis level in kidney indicated that XMU‐MP‐1 treatment did not alter fibrosis in kidney. **E)** The level of alanine aminotransferase (ALT) and **F)** creatinine kinase were determined in the serum. No significant difference was observed in serum ALT and creatinine kinase level in all groups of mice. (vehicle, N= 10; XMU‐MP‐1, N= 10).
Table S1. List of antibodies used
