# Supplementary material for: Pharmacological inhibition of Hippo pathway, with the novel kinase inhibitor XMU‐MP‐1, protects the heart against adverse effects during pressure overload
Source: Br J Pharmacol. 2019 Oct 8;176(20):3956–71. doi: 10.1111/bph.14795 (PMC6811740; doi:10.1111/bph.14795)
Supplement: Supplementary file 1 — Figure S1. Analysis of XMU‐MP‐1 effects in liver and kidney A) Representative images of liver tissue sections stained with Masson's trichrome and B) Quantification of liver fibrosis showed that there is no significant difference in liver fibrosis between vehicle treated and XMU‐MP‐1 treated mice (vehicle, N= 5; XMU‐MP‐1, N= 5). C) Images of kidney histological sections stained with Masson's trichrome. D) Assessment of fibrosis level in kidney indicated that XMU‐MP‐1 treatment did not alter fibrosis in kidney. E) The level of alanine aminotransferase (ALT) and F) creatinine kinase were determined in the serum. No significant difference was observed in serum ALT and creatinine kinase level in all groups of mice. (vehicle, N= 10; XMU‐MP‐1, N= 10). Table S1. List of antibodies used [file BPH-176-3956-s001.pdf]

Supplementary figure 1

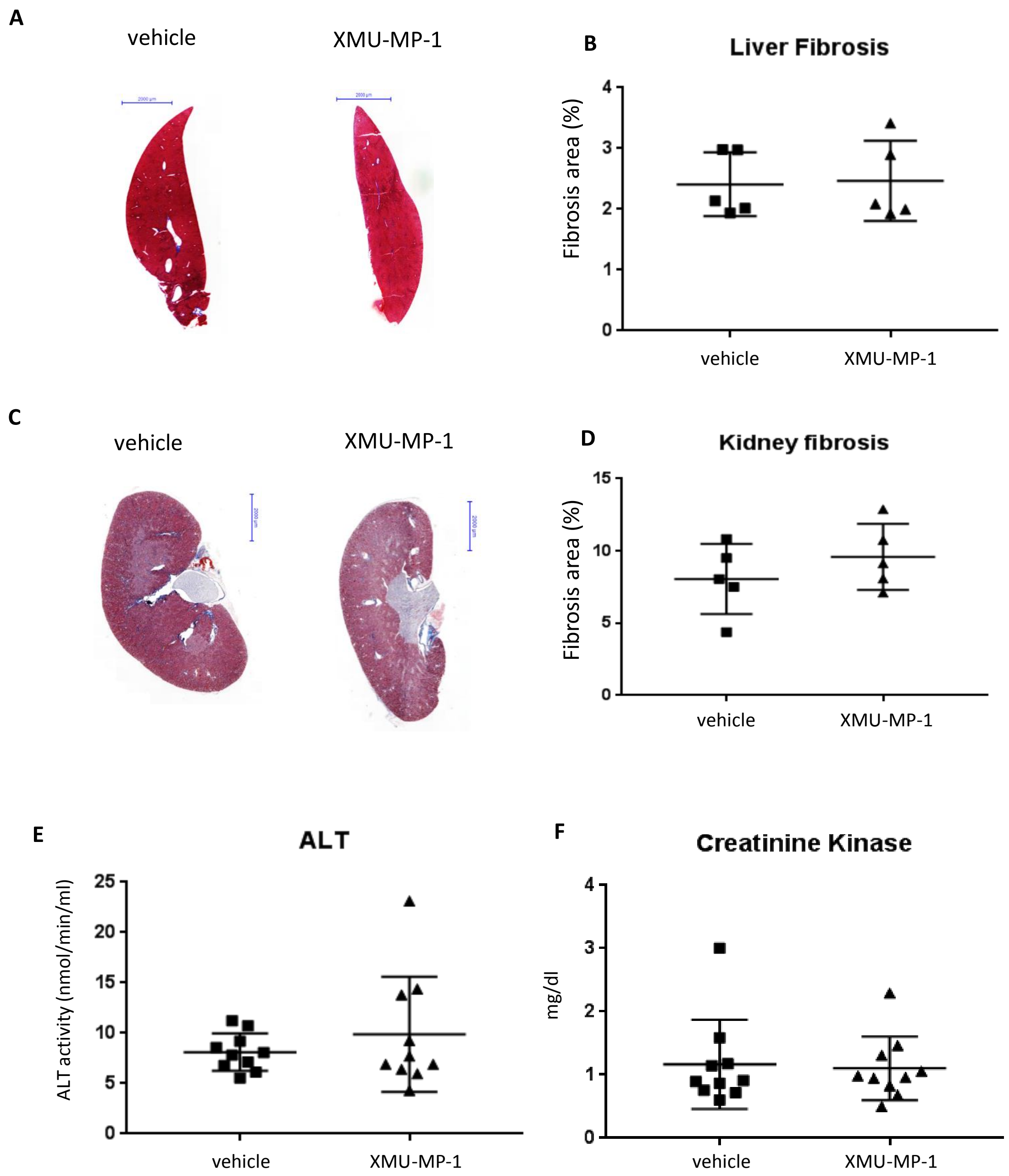

Supplementary Figure 1 Analysis of XMU-MP-1 effects in liver and kidney

A) Representative images of liver tissue sections stained with Masson's trichrome and **B)** Quantification of liver fibrosis showed that there is no significant difference in liver fibrosis between vehicle treated and XMU-MP-1 treated mice (vehicle, N= 5; XMU-MP-1, N= 5). **C)** Images of kidney histological sections stained with Masson's trichrome. **D)** Assessment of fibrosis level in kidney indicated that XMU-MP-1 treatment did not alter fibrosis in kidney. **E)** The level of alanine aminotransferase (ALT) and **F)** creatinine kinase were determined in the serum. No significant difference was observed in serum ALT and creatinine kinase level in all groups of mice. (vehicle, N= 10; XMU-MP-1, N= 10).

Supplementary Table 1 List of antibodies used

Antibody name	Antibody species	Isotype	Source	Epitope	Dilution and use
Anti-α-actinin	Mouse Monoclonal	IgG	Sigma Aldrich catalogue number A7811	Rabbit skeletal α-actinin 1	1:200 Immunofluorescence
Ki-67	Rabbit Polyclonal	IgG	Abcam catalogue number Ab15580	KLH derived from within residues 1200 - 1300 of Human Ki67	1:200 Immunofluorescence
Alexa Fluor® 647 Anti-mouse	Mouse Polyclonal	IgG	Jackson ImmunoResearch catalogue number 115-605-072	F(ab')2/Fab portion of mouse IgG	1:200 Immunofluorescence
Alexa Fluor® 488 Anti-Rabbit	Rabbit Polyclonal	IgG	Jackson ImmunoResearch catalogue number 711-545-152	Whole molecule rabbit IgG	1:200 Immunofluorescence
Non-Phospho (Active) YAP (Ser127) (E6U8Z)	Rabbit Monoclonal	IgG	Cell Signaling Technology catalogue number 29495	Endogenous YAP protein only when Ser127 is not phosphorylated (Human, Mouse, Rat, Bovine)	1:1000 Western Blot
YAP Antibody (G-6)	Mouse Monoclonal	IgG	Santa Cruz Biotechnology catalogue number sc-376830	YAP of mouse, rat and human origin	1:500 Western Blot
Phospho-MOB1 (Thr35) (D2F10)	Rabbit Monoclonal	IgG	Cell Signaling Technology catalogue number 8699	Endogenous levels of MOB1 protein only when phosphorylated at Thr35 (Human, Mouse, Rat, Monkey)	1:1000 Western Blot
MOB1 (E1N9D)	Rabbit Monoclonal	IgG	Cell Signaling Technology catalogue number 13730	total MOB1 protein (Human, Mouse, Rat, Hamster, Monkey)	1:1000 Western Blot
Anti-mouse IgG, HRP-linked Antibody	Purified horse anti-mouse IgG	IgG	Cell Signaling Technology catalogue number 7076	Mouse IgG	1:5000 Western Blot
